# ACSS2 governs milk fat synthesis in buffalo via a reciprocal positive feedback loop with SREBP1 and PPARG

**DOI:** 10.5713/ab.250642

**Published:** 2026-03-11

**Authors:** Lige Huang, Rongping Wang, Fangting Zhou, Ruixia Gao, Xinyang Fan, Yongwang Miao

**Affiliations:** 1 Faculty of Animal Science and Technology, Yunnan Agricultural University, Kunming, China; 2 Animal Genetics and Breeding Institute, Yunnan Agricultural University, Kunming, China; 3 Faculty of Animal Husbandry and Veterinary Medicine, Yunnan Vocational College of Agriculture, Kunming, China

**Keywords:** *ACSS2* Gene, Buffalo, Cell Proliferation, Milk Fat Synthesis, RNA Interference, Subcellular Localization

## Abstract

**Objective:**

Acetyl-CoA synthetase 2 (ACSS2) converts rumen-derived acetate into acetyl-CoA in ruminants. Whether it actively regulates lactation-associated transcriptional networks beyond its catalytic role remains unclear. This study aimed to characterize buffalo ACSS2 and investigate its function within the metabolic-transcriptional network of buffalo mammary epithelial cells (BuMECs).

**Methods:**

The buffalo *ACSS2* coding sequence was cloned, and its expression across lactation stages was analyzed. Subcellular localization was determined via confocal microscopy. Through siRNA-mediated knockdown in BuMECs, we assessed cell viability, triglyceride (TAG) content, and the expression of core metabolic and regulatory genes.

**Results:**

*ACSS2* expression was highly enriched in lactating mammary tissue, and the protein localized to both the nucleus and cytoplasm. In BuMECs, *ACSS2* knockdown impaired lipogenesis, significantly reducing intracellular TAG and downregulating key lipid metabolism genes (*FASN*, *ACACA*, *SCD*, *CD36*, *LPL*, *FABP3*, *DGAT1*, *DGAT2*, and *AGPAT6*). It also inhibited cell proliferation and downregulated G1/S phase regulators (*CCND1*, *CCNE1*, *CDK2*, and *CDK4*). Mechanistically, *ACSS2* depletion reduced the mRNA levels of master regulators *SREBF1* and *PPARG*, while upregulating the SREBP1-inhibitor *INSIG1*, suggesting an INSIG1-mediated blockade of the lipogenic program.

**Conclusion:**

This study establishes ACSS2 as a critical metabolic checkpoint in the buffalo mammary gland. We propose that ACSS2 maintains a reciprocal positive feedback loop with SREBP1 and PPARG. By ensuring adequate acetyl-CoA to suppress INSIG1 and support nuclear histone acetylation, ACSS2 couples substrate availability to the stability of the lipogenic program and cell cycle progression. These findings reveal a conserved metabolic-epigenetic axis essential for high-efficiency lactation in ruminants.

## INTRODUCTION

*De novo* fatty acid synthesis is a core metabolic pathway for endogenous lipid supply in mammals, and its efficiency directly dictates the synthesis rate of lipid products such as milk fat [1]. This process is highly dependent on the sufficient supply of the key metabolic precursor, acetyl-coenzyme A (acetyl-CoA). As a central metabolic node, acetyl-CoA provides the essential carbon substrate for critical enzymatic reactions catalyzed by fatty acid synthase (FASN) and acetyl-CoA carboxylase (ACACA) [2]. Consequently, the efficiency of intracellular acetyl-CoA supply is a rate-limiting factor for milk fat synthesis. A fundamental metabolic distinction exists between ruminants (cattle, goats) and monogastric animals (mice, humans) regarding the primary source of cytosolic acetyl-CoA. While monogastrics rely on ATP-citrate lyase (ACLY) to generate cytosolic acetyl-CoA from glucose [3], ruminant mammary glands exhibit negligible ACLY activity. Instead, they depend almost exclusively on the uptake of rumen-derived acetate, which is activated by acetyl-CoA synthetase 2 (ACSS2). ACSS2 is a cytosolic enzyme that directly channels acetate into the fatty acid synthesis pathway [4,5]. In mice, ACSS2 has been shown to promote systemic fat storage and utilization by selectively regulating genes involved in lipid metabolism [6]. Previous study has demonstrated that *ACSS2* expression is significantly higher in the mammary tissue of dairy cows during lactation compared to the dry period [7], underscoring its vital role in milk fat synthesis.

Based on the NCBI gene database (https://www.ncbi.nlm.nih.gov/gene/?term=), the buffalo *ACSS2* gene is located on BBU14, with three annotated transcript variants. Transcript variant X1 (XM_006048084) contains 19 exons with a coding sequence (CDS) length of 2145 bp; X2 (XM_006048085) consists of 18 exons with a CDS length of 2106 bp; and X3 (XM_044927710) contains 19 exons with a CDS length of 2055 bp. The *ACSS2* gene in domestic cattle is located on chromosome 13, with four annotated transcript variants, and its reference mRNA sequence (NM_001105339) is composed of 18 exons with a CDS of 2106 bp. This length is highly conserved among mammals: the *ACSS2* CDS in both humans (NM_018677) and mice (NM_019811) is 2106 bp, encoding 701 amino acids [8,9].

The buffalo (*Bubalus bubalis*) is an important dual-purpose livestock animal for milk and meat globally, contributing over 15% of the world’s total milk production [10]. Compared to domestic cattle (*Bos taurus*), buffalo milk has significantly higher fat, protein, and iron content, yet a markedly lower cholesterol level, giving it unique nutritional and economic value [11]. Existing buffalo breeds can generally be divided into two types: the swamp buffalo (2n = 48), primarily used for draft purposes, and the river buffalo (2n = 50), known for its high milk production performance [12,13]. Among them, the Binglangjiang buffalo, as the only indigenous river-type breed in Yunnan, China, serves as an ideal animal model for studying the molecular mechanisms of high milk fat formation due to its excellent milk production performance. Recent studies in mice have highlighted ACSS2’s role in epigenetic regulation under metabolic stress [14]. Unlike monogastric species where glucose-derived citrate is the primary lipogenic precursor, ruminants rely almost exclusively on acetate absorbed from the rumen. This places ACSS2 at the apex of the lipogenic hierarchy. While *ACSS2* is known to be regulated by SREBP1, it remains unknown whether ACSS2 exerts feedback control over these master regulators in an acetate-dependent system like the buffalo. Therefore, elucidating the specific mechanism of ACSS2 in buffalo milk fat synthesis is of significant scientific importance. To address this knowledge gap, this study first obtained the complete CDS of the buffalo *ACSS2* gene through molecular cloning and systematically analyzed its molecular characteristics using bioinformatics and comparative genomics. Subsequently, real-time quantitative polymerase chain reaction (qPCR) was used to detect the expression of *ACSS2* in different tissues and buffalo mammary epithelial cells (BuMECs). Finally, using BuMECs as an *in vitro* model, the molecular network involving ACSS2 in buffalo milk fat synthesis was thoroughly investigated to lay a theoretical foundation for improving the quality of buffalo milk fat.

## MATERIALS AND METHODS

### Sample collection

All experimental animals were sourced from the Binglangjiang Buffalo Conservation Farm in Tengchong City, Yunnan, and were raised under standardized conditions. Each buffalo underwent a clinical veterinary examination to confirm the absence of mastitis and other systemic diseases. The minimum number of animals required to meet the experimental objectives was used, and all effective measures were taken to minimize their suffering.

Ten healthy female Binglangjiang buffaloes of similar parity and age (36±2 months old) were selected for the experiment, with five at peak lactation (60 days postpartum) and five in the dry period (non-pregnant, 60 days prepartum). After slaughter, tissue samples from the heart, liver, spleen, lung, kidney, mammary gland, muscle, rumen, brain, and small intestine were immediately collected, flash-frozen in liquid nitrogen, and then transferred to a −80°C ultra-low temperature freezer for subsequent RNA extraction. All analyses were performed under blinded conditions, meaning the researchers preparing the samples and analyzing the data were unaware of the group assignments.

### Isolation and identification of the *ACSS2* gene

Total RNA was isolated using the RNAiso Plus reagent kit (TaKaRa) according to the manufacturer’s instructions. RNA integrity was assessed by 1.5% agarose gel electrophoresis, and its concentration (A_260_) and purity (A_260_/A_280_ ratio) were determined using a NanoDrop 2000 UV-Vis spectrophotometer (Thermo Fisher Scientific). First-strand cDNA was synthesized from 2–3 μg of RNA using a reverse transcription kit (TaKaRa) with Oligo(dT)_18_ (500 μg/mL) as the primer.

Based on the buffalo *ACSS2* mRNA sequence (XM_ 044927710) from the NCBI database, specific primers for isolating the *ACSS2* CDS were designed using Primer Premier 5.0 software [15] (Supplement 1). The 10 μL PCR reaction system included 0.5 μL each of forward and reverse primers (10 μmol/L), 5 μL of 2×Es Taq Master Mix (CWBIO), 1 μL of cDNA template (200 ng/μL), and 3 μL of ddH_2_O. The PCR program was as follows: pre-denaturation at 94°C for 5 minutes, followed by 35 cycles of denaturation at 94°C for 30 seconds, annealing at 59.8°C for 30 seconds, and extension at 72°C for 2 minutes, with a final extension at 72°C for 7 minutes, and termination at 4°C. The PCR product was visualized by 1.5% agarose gel electrophoresis, and the target band was purified and cloned into the pMD-18T vector (TaKaRa). To identify the transcript variants of *ACSS2* in the buffalo mammary gland, 20 positive single colonies were selected for liquid culture and subjected to bidirectional sequencing by Sangon Biotech.

The obtained sequences were checked and assembled using the Seqman software within the Lasergene package (DNAStar). The Open Reading Frame (ORF) was identified using the ORF Finder program (https://www.ncbi.nlm.nih.gov/orffinder/), and the corresponding amino acid sequence was deduced using EditSeq software (DNAStar). The resulting CDS was used as a query sequence for a homology search via the BLAST program (https://blast.ncbi.nlm.nih.gov/Blast.cgi) to confirm it was the target sequence.

### Bioinformatics and comparative genomics analysis

A comprehensive analysis of the buffalo ACSS2 protein was conducted using various online tools. The physicochemical properties, signal peptide, transmembrane domains, and functional modification sites of ACSS2 were analyzed using ProtParam (https://web.expasy.org/protparam/), SignalP 5.0 (https://services.healthtech.dtu.dk/services/SignalP-5.0/), TMHMM-2.0 (https://services.healthtech.dtu.dk/service.php?TMHMM-2.0), and PrositeScan (https://prosite.expasy.org/prosite.html), respectively. Secondary and tertiary structure predictions were performed using SOPMA (https://npsa-pbil.ibcp.fr/cgi-bin/npsa_automat.pl?page=/NPSA/npsa_sopma.html) and SWISS-MODEL (https://swissmodel.expasy.org/), respectively. Subcellular localization and protein interactions were predicted using ProtComp (http://www.softberry.com/berry.phtml?topic=protcompan&group=programs&subgroup=proloc) and STRING (https://cn.string-db.org/). DAVID (https://davidbioinformatics.nih.gov/) was used for the analysis of biological processes and molecular functions.

The amino acid sequences of ACSS2 from buffalo, cattle, yak, zebu, bison, goat, sheep, horse, camel, deer, rat, and human were downloaded from the NCBI database (Supplement 2). Structural information of the *ACSS2* transcription region was derived from the genomic annotation feature files in the NCBI database. The complete structure of the transcription region was assembled using the GXF function of TBtools software and visualized with Gene Structure Display Server 2.0. The conserved motif composition of ACSS2 was determined using the MEME online tool. The conserved domains of ACSS2 were analyzed using the NCBI Batch Web CD-Search Tool. A phylogenetic tree was constructed based on the ACSS2 amino acid sequences using the maximum likelihood method with the Jones-Taylor-Thornton (JTT)+Gamma (G) model in MEGA7 software [16], and a bootstrap test with 5,000 replicates was performed. The phylogenetic tree, motifs, and conserved domains were integrated using TBtools software.

### Cell culture

BuMECs were isolated and purified from the mammary tissue of buffalo at peak lactation according to a previously established method by our group [17]. Cells were cultured at 37°C in a 5% CO_2_ environment. The culture medium was DMEM/F12 (Gibco) supplemented with 5 μg/mL insulin (Sigma-Aldrich), 1 μg/mL penicillin-streptomycin (Sigma-Aldrich), 10 ng/mL epidermal growth factor (Sigma-Aldrich), 1 μg/mL hydrocortisone (Sigma-Aldrich), and 10% fetal bovine serum (Gibco). Before experiments, BuMECs were cultured in an induction medium containing 3 ng/μL prolactin (Sigma-Aldrich) for 48 h to prepare for subsequent experiments.

### Subcellular localization analysis

The CDS of *ACSS2* obtained in this study was inserted into the pEGFP-N1 vector to construct the overexpression plasmid pEGFP-N1-*ACSS2*. The recombinant plasmid was transfected into BuMECs to analyze the subcellular localization of the ACSS2 protein. The cell mitochondria and nuclei were stained using MitoTracker Red CMXRos (Solarbio) and DAPI solution (Solarbio), respectively, following the kit instructions. After staining, the staining solution was removed, and the cells were observed under a laser scanning confocal microscope (Olympus).

### Small interfering RNA transfection

Two pairs of siRNAs targeting different regions of the *ACSS2* CDS (siRNA1-*ACSS2* and siRNA2-*ACSS2*), along with a negative control siRNA (siRNA-NC) with no homology to any genes in BuMECs, were designed and synthesized by Shanghai Sangon Biotech (Supplement 1). When cell confluence reached 80%, the siRNAs targeting *ACSS2* were transfected into the cells using TransIntro EL transfection reagent (TransGen Biotech) according to the manufacturer’s instructions to screen for the siRNA with the highest interference efficiency. Subsequently, the most effective siRNA-*ACSS2* and the corresponding siRNA-NC were transfected into the cells. The medium was replaced with basic culture medium 4–6 h post-transfection. Cells were collected 48 h post-transfection for RNA extraction, cell viability assay, and triglyceride (TAG) content measurement. Each experiment was performed with three biological replicates, and each biological replicate included three technical replicates.

### Real-time quantitative polymerase chain reaction

RNA was extracted from tissues and cells, and first-strand cDNA was synthesized as described in section 2.2. The qPCR experiments were performed using dib SYBR qPCR SuperMix Plus (Aibisheng) on a LineGene 9600 system (BIOER). The preparation of the qPCR reaction system and the reaction program followed the manufacturer’s instructions. The qPCR products were confirmed by Sanger sequencing and BLAST homology alignment to ensure the amplified fragments were the target genes. The specificity of the PCR products was verified by melting curve analysis, and the qPCR amplification efficiency was determined using the LinRegPCR program. Each qPCR detection was conducted in three independent experiments, with each experiment having three technical replicates to ensure the reliability of the results. The mRNA expression levels of the target genes were normalized using *ACTB*, *GAPDH*, and *RPS23* as internal reference genes, and the relative mRNA abundance was calculated using the 2^−ΔΔCT^ method. The qPCR primer sequences are listed in Supplement 1.

### Cell viability assay

The viability of BuMECs was measured using the Cell Counting Kit-8 (CCK-8) assay. BuMECs were seeded in 96-well plates at a density of 2×10^4^ cells/well and incubated overnight at 37°C in a 5% CO_2_ incubator. After cell attachment, they were treated with siRNA-*ACSS2* and siRNA-NC transfection and cultured for another 48 h. Subsequently, 10 μL of CCK-8 solution (Shangwei) was added to each well and incubated at 37°C in the dark for 2 h. After shaking for 15 minutes at 500 rpm on a micro-oscillator, the absorbance at a wavelength of 450 nm was measured using a Varioskan Lux multi-function microplate reader (Thermo Fisher Scientific).

### Measurement of intracellular total triglyceride

To quantify the intracellular TAG levels, cells transfected with siRNA-*ACSS2* and siRNA-NC were washed twice with pre-cooled PBS and then assayed using a Tissue/Cell Triglyceride Assay Kit (PPLY) according to the manufacturer’s instructions. Concurrently, the total protein concentration was determined using a BCA Protein Assay Kit (Beyotime). The TAG content was calculated by normalizing to the total cellular protein concentration and is reported as μM/g protein.

### Data analysis

Experimental data are presented as the mean±standard error of the mean (mean±SEM) from three independent biological replicates. A two-tailed unpaired Student’s t-test was performed using GraphPad Prism 5 software (GraphPad Software) to analyze the mean differences between two groups. A p-value<0.05 was considered statistically significant (* p<0.05, ** p<0.01, *** p<0.001).

## RESULTS

### Cloning, identification, and molecular characterization of buffalo *ACSS2*

Based on single-colony screening and Sanger sequencing, a single transcript of the buffalo *ACSS2* gene was successfully isolated (Supplement 3). This transcript has a CDS of 2106 bp (NCBI Accession No.: KJ472108) and encodes a polypeptide of 701 amino acids (Supplement 4). A BLASTn alignment analysis showed that this sequence has 100% identity with the CDS of the predicted buffalo *ACSS2* transcript variant X2 (XM_006048085.2) in the NCBI database, thus confirming it as the buffalo *ACSS2* gene. Bioinformatics analysis revealed that the buffalo ACSS2 protein is a hydrophilic protein with a molecular weight of approximately 78.6 kDa, containing no signal peptide or transmembrane domains, but possessing five types of functional modification sites (Supplements 5, 6). Its basic physicochemical properties are highly similar to those of ACSS2 in other bovid species (Supplement 5).

### The *ACSS2* gene is highly conserved among mammals

**Figure 1:**
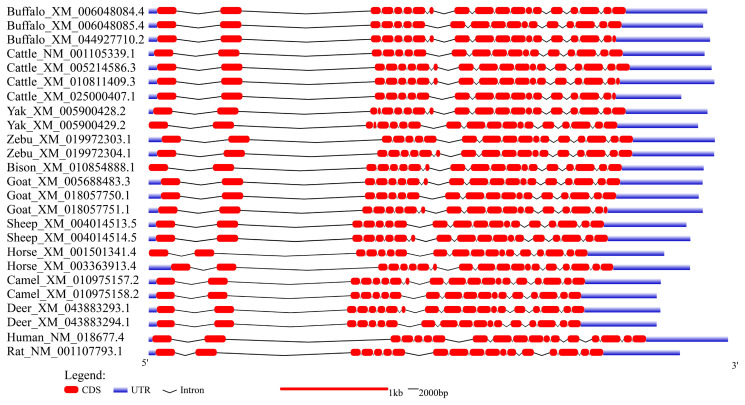
Transcriptional structure of *ACSS2* in buffalo and other species. CDS, coding sequence; UTR, untranslated region; *ACSS2*, acetyl-CoA synthetase 2.

To evaluate the evolutionary conservation of ACSS2, this study conducted a cross-species comparative analysis of its transcript structure and encoded protein sequence. The results showed that the exon-intron arrangement of buffalo *ACSS2* is highly similar to that of other bovid species (Figure 1 and Supplement 7). Its CDS length of 2106 bp and encoded protein of 701 amino acids are highly conserved among mammals. Amino acid sequence alignment indicated that the buffalo ACSS2 protein shares a high sequence identity of 97.5%–99.3% with its homologous proteins from other bovid species (Supplement 8). Phylogenetic analysis showed that the buffalo clusters into a single branch with *Bos* species, indicating the closest evolutionary relationship (Supplement 9A). All analyzed species contain a conserved acetyl-CoA synthetase (ACS) domain and 10 conserved motifs (Supplements 9B, 9C); this domain is the core functional unit for catalyzing the formation of acetyl-CoA from acetate. Furthermore, the secondary structure composition (Supplements 10, 11) and tertiary structure (Supplement 12) of buffalo ACSS2 are also highly consistent with those of other bovid species. Although CDSs and domains exhibit high conservation, differences in the length and nucleotide composition of 5’- and 3’-untranslated regions (UTRs) across species suggest the existence of species-specific post-transcriptional regulatory mechanisms. In summary, these findings reveal strong conservation of the CDS and core structure of buffalo ACSS2, while also highlighting species-specific differences in UTR architecture.

### Differential gene expression

**Figure 2:**
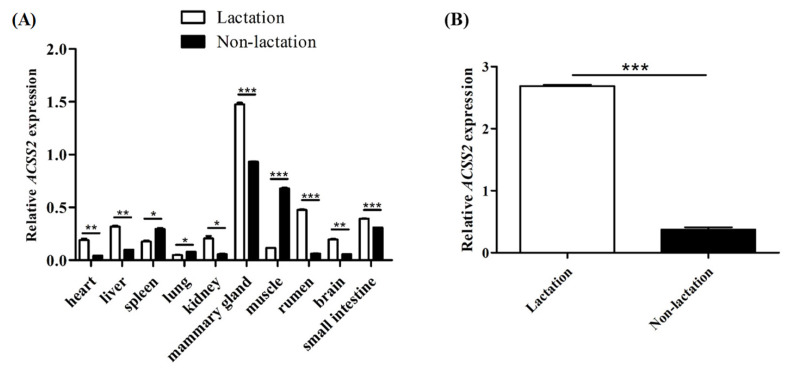
Expression of the buffalo *ACSS2* gene in various tissues and cells. (A) Relative mRNA expression of *ACSS2* in tissues from lactating and non-lactating buffalo. (B) Relative mRNA expression of *ACSS2* in BuMECs from lactating and non-lactating states. Data are presented as the mean±SEM (n = 3). Asterisks indicate statistical significance (* p<0.05, ** p<0.01, *** p<0.001). *ACSS2*, acetyl-CoA synthetase 2; BuMECs, buffalo mammary epithelial cells; SEM, standard error of the mean.

qPCR analysis revealed that the *ACSS2* gene was expressed in all 10 buffalo tissues examined (Figure 2A). In both physiological states, *ACSS2* expression was highest in the mammary gland. During lactation, the next highest levels were in the rumen, small intestine, and liver (p<0.05), whereas during the non-lactating period, they were in muscle, small intestine, and spleen (p<0.05). Notably, *ACSS2* expression in the mammary gland was higher during lactation compared to the non-lactating period (p<0.001). This tissue-level finding was corroborated at the cellular level, where *ACSS2* expression in lactating-state cells was also significantly higher than in non-lactating-state cells (p<0.001, Figure 2B).

### Subcellular localization, biological processes, and molecular functions

**Figure 3:**
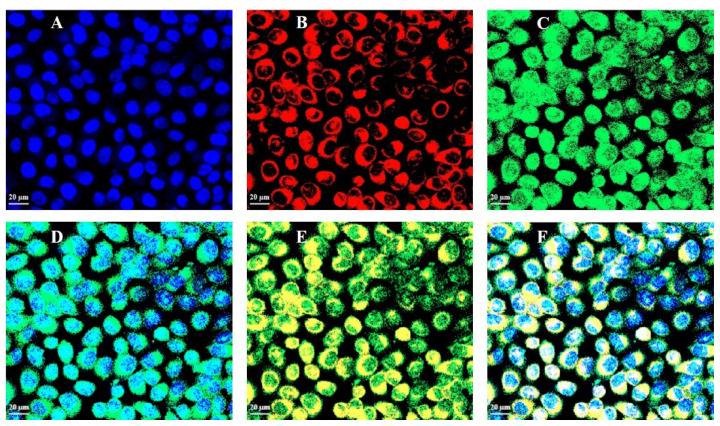
Nuclear-cytoplasmic localization of ACSS2 in BuMECs. (A) Nuclei stained with DAPI. (B) Mitochondria stained with MitoTracker. (C) EGFP-*ACSS2* expressed as green fluorescent protein. (D) Merge overlay of EGFP-*ACSS2* and nuclei. (E) Merge image of mitochondria and EGFP-*ACSS2*. (F) Merge image of nuclei, mitochondria, and EGFP-*ACSS2*. ACSS2, acetyl-CoA synthetase 2; BuMECs, buffalo mammary epithelial cells.

To investigate its location of action, the distribution of the EGFP-*ACSS2* fusion protein in BuMECs was observed using a confocal microscope. The results showed that the green fluorescent signal of EGFP-*ACSS2* overlapped with the red fluorescence marking the cytoplasm, whilst also overlapping with the blue fluorescence marking the nucleus (Figure 3). Consistent with its dual metabolic and epigenetic roles observed in other mammals, ACSS2 exhibited a nucleocytoplasmic distribution [14,18]. Functional enrichment analysis revealed that buffalo ACSS2 possesses acetate-CoA ligase and propionate-CoA ligase activities and participates in key biological processes such as acetyl-CoA biosynthesis and lipid metabolism. The experimental results indicate that the ACSS2 protein plays a dual functional role in both the cytoplasm and nucleus of BuMECs.

### *ACSS2* knockdown inhibits the proliferation of buffalo mammary epithelial cells

**Figure 4:**
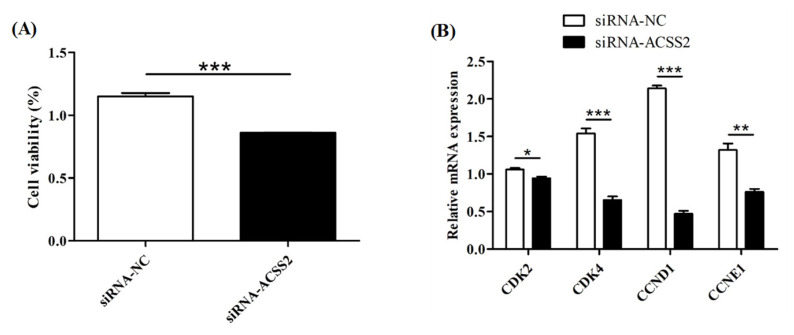
Effect of *ACSS2* on the proliferation of BuMECs. (A) Effects of *ACSS2* knockdown on cell viability. (B) Effects of *ACSS2* knockdown on the expression of cell cycle-related genes. The values are expressed as mean±SEM (n = 3); * p<0.05, ** p<0.01, *** p<0.001. *ACSS2*, acetyl-CoA synthetase 2; BuMECs, buffalo mammary epithelial cells; SEM, standard error of the mean.

To explore whether ACSS2 affects the proliferation of BuMECs, this study used siRNA technology to knock down the mRNA level of *ACSS2*. Compared to the control group (siRNA-NC), siRNA1-*ACSS2* and siRNA2-*ACSS2* reduced the mRNA expression level of *ACSS2* in BuMECs by 75.6% and 56.8%, respectively (Supplement 13). Since siRNA1-*ACSS2* had the optimal interference efficiency, it was selected for subsequent functional experiments. The CCK-8 assay results showed that, compared to the control group, knocking down *ACSS2* significantly inhibited the cell viability of BuMECs (p<0.001, Figure 4A). To further investigate the molecular mechanism, the expression of key cell cycle regulators was examined (Figure 4B). The results indicated that *ACSS2* knockdown significantly downregulated the mRNA levels of *CDK2* (p<0.05), *CDK4* (p<0.001), *CCND1* (p<0.001), and *CCNE1* (p<0.01). These results suggest that ACSS2 promotes the proliferation of mammary epithelial cells by maintaining the expression of positive cell cycle regulators.

### *ACSS2* knockdown inhibits milk fat synthesis in buffalo mammary epithelial cells

**Figure 5:**
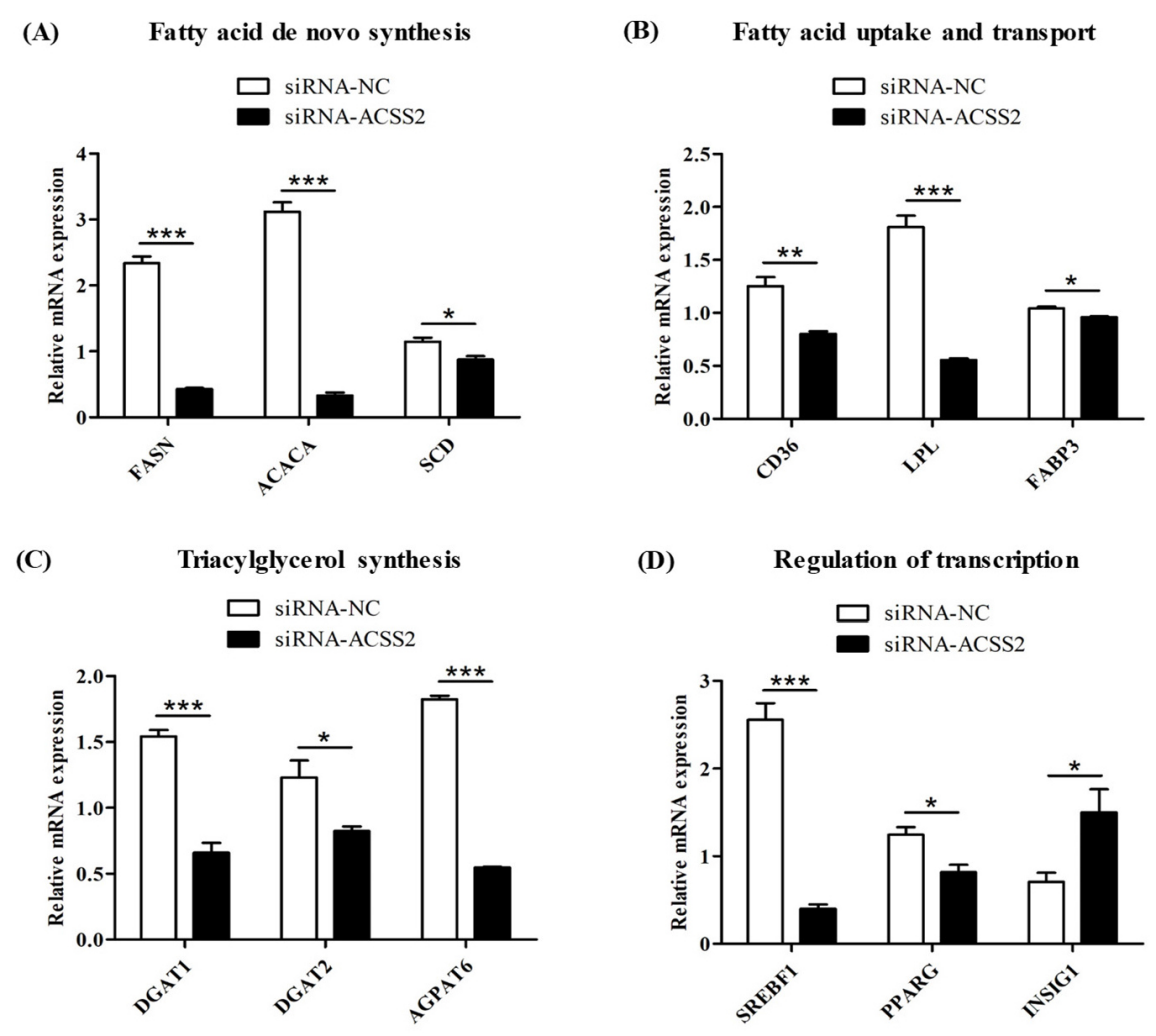
Effects of *ACSS2* knockdown on genes related to milk fat synthesis in BuMECs. (A) Changes in genes related to *de novo* fatty acid synthesis. (B) Alterations in genes associated with fatty acid uptake and transport. (C) Changes in genes involved in TAG synthesis. (D) Alterations in transcription regulation-related genes. Data are presented as mean±SEM (n = 3); * p<0.05, ** p<0.01, *** p<0.001. *ACSS2*, acetyl-CoA synthetase 2; BuMECs, buffalo mammary epithelial cells; TAG, triglyceride; SEM, standard error of the mean.

**Figure 6:**
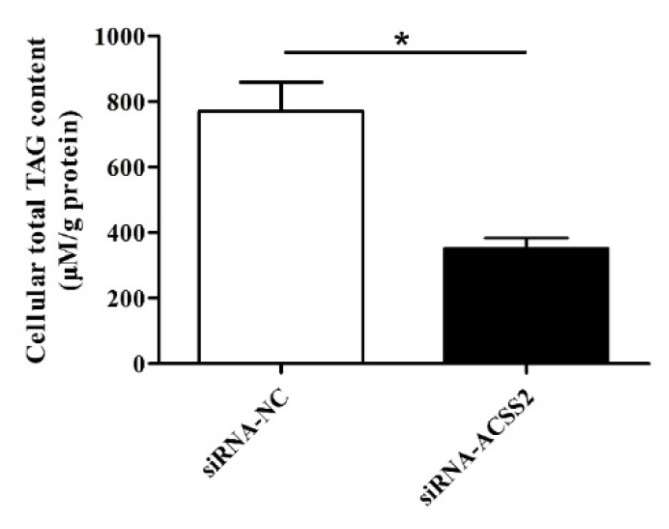
Effect of *ACSS2* gene knockdown on TAG content. Values are presented as mean±SEM (n = 3). Statistical significance is expressed as follows: * p<0.05. TAG, triglyceride; *ACSS2*, acetyl-CoA synthetase 2; SEM, standard error of the mean.

To elucidate the function of ACSS2 in BuMECs milk fat metabolism, this study systematically evaluated the expression changes of key milk fat metabolism genes via qPCR following siRNA-mediated *ACSS2* knockdown (Figure 5). The results showed that genes related to *de novo* fatty acid synthesis (*FASN*, *ACACA*, *SCD*) and fatty acid uptake/transport (*CD36*, *LPL*, *FABP3*) were all significantly downregulated (p<0.05, with *FASN*, *ACACA*, and *LPL* at p<0.001). TAG synthesis genes *DGAT1*, *DGAT2*, and *AGPAT6* were also synchronously decreased (*DGAT1*, *AGPAT6*: p<0.001; *DGAT2*: p<0.05). At the transcriptional level, the positive regulators of milk fat synthesis, *SREBF1* and *PPARG*, were significantly downregulated (*SREBF1*: p<0.001; *PPARG*: p<0.05), while the mRNA abundance of the negative regulator *INSIG1* was significantly upregulated (p<0.05). Consistent with these findings, *ACSS2* knockdown significantly reduced the TAG content within BuMECs (p<0.05; Figure 6). Strikingly, while positive regulators (*SREBF1*, *PPARG*) were downregulated, the negative regulator *INSIG1* was significantly upregulated. This provides a mechanistic clue that intracellular acetyl-CoA levels may modulate the SREBP1 processing pathway.

## DISCUSSION

ACSS2 is a pivotal enzyme that catalyzes the formation of acetyl-CoA, a fundamental precursor for *de novo* fatty acid synthesis. ACSS2 was initially identified in rat liver as a cytosolic enzyme involved in fatty acid synthesis [19,20] and was confirmed to be a target gene of SREBP in mice [21]. Unlike murine models where ACLY dominates, the widespread and lactation-induced expression of *ACSS2* in buffalo reflects the species’ evolutionary adaptation to acetate utilization. Our study confirms ACSS2 as the primary gatekeeper of lipogenesis in buffalo.

The cloned CDS of the buffalo *ACSS2* gene transcript is 2106 bp long and encodes a polypeptide of 701 amino acids. The *ACSS2* coding region is highly conserved across species, while differences were observed in the 5’-UTR and 3’-UTR of the mRNA structure. At the amino acid level, buffalo ACSS2 exhibits nearly complete identity with its homologs in other bovids, with the core catalytic motif fully conserved, underscoring its fundamental role as an acetyl-CoA synthetase in cellular metabolism [22]. This conservation implies a shared mechanism for acetyl-CoA production—a key precursor in biosynthesis pathways such as fatty acid synthesis. Given the high conservation of the protein sequence, the observed species-specific differences in *ACSS2* expression levels may stem from the variations we identified in the UTRs, which are known to influence translational efficiency and mRNA stability [23,24]. The species-specific differences in UTR structure and sequence could therefore fine-tune transcript abundance and translational efficiency in mammary epithelial cells, contributing to the stronger induction of ACSS2 and the high milk fat synthesis observed in buffalo.

The tissue distribution and lactation-associated expression pattern of *ACSS2* in buffalo also reveal conservation across species. This study found that *ACSS2* is expressed in all 10 tested tissues of the buffalo, consistent with its widespread multi-tissue expression in dairy goats [25], indicating its role as a basal metabolic enzyme involved in the regulation of systemic lipid and energy metabolism. However, its expression level shows significant tissue-specific differences during lactation: the expression of *ACSS2* in the heart, liver, kidney, mammary gland, rumen, brain, and small intestine is significantly upregulated during lactation, whereas it is downregulated in the spleen, lung and muscle tissues. This inter-tissue expression difference reflects the classic “synthesis-first” energy partitioning strategy of ruminants during lactation [26]. Consistent with the enhanced utilization of acetate by the liver to synthesize lipid precursors in lactating ruminants [27], the upregulation of *ACSS2* in the buffalo liver aims to provide sufficient precursors for milk fat synthesis in the mammary gland. The downregulation in muscle is likely a mechanism for the organism to preferentially allocate limited energy to metabolically active organs such as the mammary gland. Consistent with findings in dairy cows, where *ACSS2* is up-regulated in mammary tissue during lactation [7], we observed that *ACSS2* expression increased markedly at peak lactation compared with the dry period. Since buffalo milk generally contains higher fat levels and more short- and medium-chain fatty acids than bovine milk [28], the induction of *ACSS2* during buffalo lactation may reflect stronger acetate-dependent lipid synthesis in buffalo mammary glands. Thus, while the general pattern of mammary-enriched and lactation-induced *ACSS2* expression appears to be conserved across ruminants, its magnitude and potential contribution to milk fat yield may differ between buffalo and cattle.

**Figure 7:**
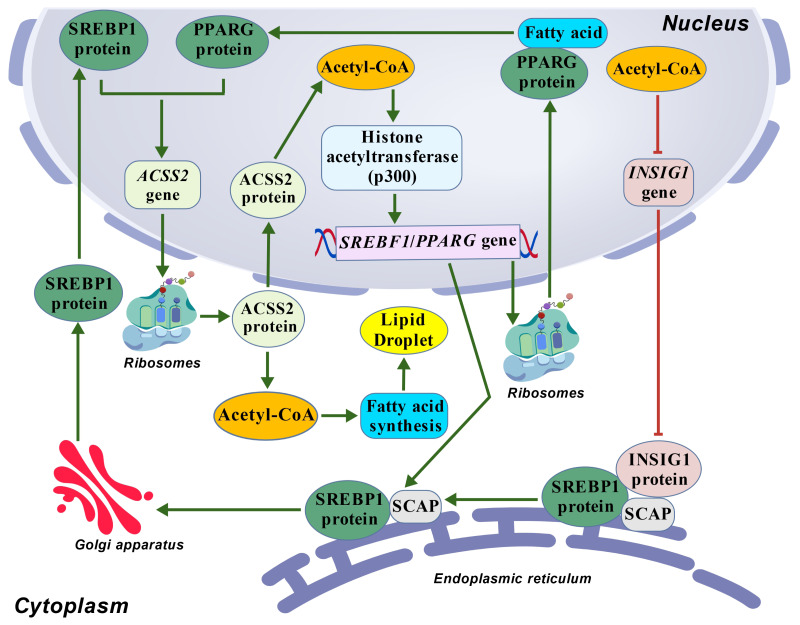
ACSS2-mediated dual positive metabolic-epigenetic feedback loop in BuMECs. “→” represents activation, and “⊣” represents inhibition. Although inhibitory component (INSIG1) is present, the overall circuit functions as a net positive feedback loop. ACSS2, acetyl-CoA synthetase 2; BuMECs, buffalo mammary epithelial cells.

The most insightful finding of this study is the discovery that ACSS2 is not just a downstream target but an active participant in a reciprocal positive feedback loop with its master transcriptional regulators, SREBP1 and PPARG (Figure 7). This finding fundamentally revises the understanding of the lipogenic regulatory network from a simple linear cascade to a dynamic, self-reinforcing circuit. The “forward” arm of this circuit is well-established: SREBP1 and PPARG are core transcription factors that directly bind to the promoters of lipogenic genes, including *ACSS2*, to activate their transcription—a relationship previously documented in BuMECs, where *PPARG* overexpression drives *ACSS2* upregulation [29], and in other mammals where SREBP1 regulates *ACSS2* expression has been reported [25]. However, this study provides compelling evidence for a “reverse” arm, where the metabolic output of ACSS2 is required to sustain the activity of SREBP1 and PPARG. The mechanism for SREBP1 regulation appears to be mediated by the metabolic sensor INSIG1. SREBP1 is synthesized as an inactive precursor tethered to the endoplasmic reticulum (ER) membrane. Its activation requires it to be transported to the Golgi for proteolytic cleavage, a step that is inhibited when INSIG1 binds to the SREBP1-SCAP complex, retaining it in the ER [30]. This study demonstrates that *ACSS2* knockdown leads to a significant increase in *INSIG1* mRNA levels. We hypothesize that the acute depletion of acetyl-CoA following *ACSS2* knockdown signals a state of “metabolic starvation”, which triggers the compensatory upregulation of INSIG1 (possibly via an ER stress response). The accumulation of INSIG1 functionally locks SREBP1 in the ER, preventing its nuclear entry. Since nuclear SREBP1 is known to bind its own promoter to maintain its expression (auto-regulation), this blockade leads to the observed downregulation of *SREBF1* mRNA. The activity of SREBP itself can be influenced by the availability of acetyl-CoA for the proper cleavage, maturation, and activation of the SREBP1 precursor protein [31], which can stabilize the protein and enhance its transcriptional activity. Thus, ACSS2 maintains the lipogenic program not only by providing substrate (acetyl-CoA) but also by repressing the inhibitor INSIG1 to keep the SREBP1 activation pathway open. The regulation of PPARG by ACSS2 is likely indirect but equally critical. PPARG is a nuclear receptor whose transcriptional activity is dependent on binding to endogenous ligands, primarily fatty acids and their derivatives [32]. By crippling the supply of acetyl-CoA, *ACSS2* knockdown directly inhibits *de novo* fatty acid synthesis, as evidenced by the sharp downregulation of *FASN* and *SCD*. This reduction in the synthesis of endogenous fatty acids would logically deplete the pool of natural PPARG ligands, leading to decreased PPARG activation and a subsequent reduction in the transcription of its target genes. This “downstream feeding back to upstream” mechanism constitutes a self-reinforcing positive feedback system: high levels of SREBP1 and PPARG drive high levels of *ACSS2* expression, and the acetyl-CoA produced by the latter provides the metabolic basis for maintaining the high activity of SREBP1 and PPARG. This suggests that, although the individual components of the lipogenic regulatory network are conserved, the quantitative wiring and feedback strength of this network may differ between species. The reinforced ACSS2–SREBP1–PPARG axis could thus contribute to the high milk fat content and efficient acetate utilization observed in buffalo [33] to ensure robust milk fat synthesis under fluctuating metabolic conditions.

Our observation of ACSS2 in the nucleus of BuMECs parallels the recent findings by Zhang et al [14], who reported that ACSS2 translocates to the nucleus in murine *β*-cells during pregnancy to mediate histone acetylation at specific loci. This suggests an evolutionary conservation among mammalian secretory tissues (pancreatic islets vs. mammary glands) utilize the same metabolic enzyme—ACSS2—as an epigenetic switch to couple substrate availability (acetate) with the high-demand gene expression program required for lactation or insulin secretion. We propose that a similar “metabolic-epigenetic axis” also governs lactation physiology in buffalo. In our study, the downregulation of gene expression, including genes related to the cell cycle, lipid metabolism, and even the transcription factors themselves, after *ACSS2* knockdown may not be solely an indirect consequence of reduced cytoplasmic metabolites. A more direct functional mechanism could be that BuMECs are in an enhanced state of gene transcriptional activity during the peak lactation period, while the absence of *ACSS2* leads to a “nuclear acetyl-CoA famine”, causing a decrease in the level of activating histone acetylation marks (such as H3K27ac, H3K9ac) at the promoter regions of these key genes. In ruminant mammary glands, acetate rather than glucose is the primary carbon source for *de novo* fatty acid synthesis [34], which may enhance the functional importance of ACSS2-mediated acetate activation in both cytoplasmic and nuclear compartments. The nuclear ACSS2 in BuMECs therefore suggests that, while the subcellular distribution of ACSS2 is conserved with that in non-ruminant species, its role in coupling acetate utilization to the transcriptional control of genes may be particularly critical in acetate-dependent species such as buffalo.

This study confirms that the knockdown of *ACSS2* delivered a dual blow to BuMECs. First, at the level of milk fat synthesis, a significant decrease in the total intracellular TAG content was observed. This decline is the result of two contributing factors: one is the direct substrate scarcity, i.e., the reduced supply of acetyl-CoA, which directly limits the rate of *de novo* fatty acid synthesis; the other is the suppressed activity of the entire SREBP1/PPARG transcriptional network for lipid synthesis, leading to a comprehensive downregulation of the mRNA levels of all its key downstream effector genes, including those for fatty acid synthesis (*FASN*, *ACACA*, *SCD*), transport (*CD36*, *LPL*, *FABP3*), and TAG synthesis (*DGAT1*, *DGAT2*, *AGPAT6*). Second, at the level of cell proliferation, the knockdown of *ACSS2* significantly inhibited cell viability and was accompanied by the downregulation of core cell cycle machinery, including the G1/S phase regulators *CCND1/CDK4* and *CCNE1/CDK2* [35,36], suggesting that ACSS2-derived acetyl-CoA is rate-limiting for the G1/S transition in BuMECs*.* This aligns with the “metabolic checkpoint” theory, where sufficient cytosolic/nuclear acetyl-CoA is required to acetylate histones at cyclin gene promoters, thereby licensing cell division only when metabolic resources are abundant [37–39]. The metabolic stress induced by *ACSS2* deficiency appears to be sufficient to halt cell cycle progression, preventing the expansion of the functional mammary epithelial cell population. In line with studies where ACSS2 supports lipid synthesis and cell survival by supplying acetyl-CoA for fatty acid and cholesterol biosynthesis under nutrient-limiting or hypoxic conditions [40]. Importantly, the critical role of acetate flux and mammary metabolism in determining milk output is underscored by recent applied research. A study on Anatolian buffalo demonstrated that supplementary feeding (particularly with mixed rations containing roughage) significantly improved milk yield and fat content, which likely increases the supply of lipogenic precursors, particularly rumen-derived acetate, to the mammary gland [41]. Our findings provide a molecular framework to explain these production-level observations: the enhanced acetate supply would be activated by ACSS2, not only fueling cytoplasmic lipogenesis but also, via its nuclear role and the reinforcement of the SREBP1/PPARG transcriptional loop, sustaining the high transcriptional activity necessary for efficient milk fat synthesis. Thus, our results support a conserved role of ACSS2 as a metabolic node sustaining mammary lipogenesis across mammalian species, and reveal that the integrity of this regulatory circuit is crucial for maintaining both the cell number and secretory activity of the mammary tissue during lactation.

While our study provides robust evidence for a functional genetic interaction between ACSS2 and the SREBP1/PPARG network, we acknowledge a technical limitation: we did not perform ChIP-seq or reporter assays to map direct chromosomal binding sites. This was due to the current lack of validated, high-specificity antibodies for buffalo transcription factors. Consequently, we cannot rule out that the regulation of *SREBF1* and *PPARG* mRNA levels is entirely indirect, mediated solely by metabolite sensing (via INSIG1) rather than physical promoter occupancy by ACSS2. Future studies utilizing emerging proteomic tools for non-model organisms will be essential to map the precise chromatin landscape of this feedback loop.

## CONCLUSION

This study systematically elucidated the molecular characteristics of buffalo ACSS2, and revealed its dual core role in the regulation of milk fat synthesis. We propose the existence of a “functional reciprocal positive feedback loop” between ACSS2 and the SREBP1/PPARG transcriptional network: by ensuring a stable supply of the key metabolite acetyl-CoA, ACSS2, in turn, supports the sustained activation of its upstream transcriptional activators, thereby guaranteeing the robust operation of the milk fat synthesis program. Concurrently, the phenomenon of ACSS2 nuclear localization puts forward a scientific hypothesis that it may participate in epigenetic regulation by modulating histone acetylation, pointing to a new direction for future research. These findings deepen our understanding of the molecular mechanisms of lactation in ruminants and provide a solid theoretical basis and a highly potential molecular target for improving the milk quality of buffalo by regulating key metabolic-transcriptional hubs.

## Data Availability

Upon reasonable request, the datasets of this study can be available from the corresponding author.

## SUPPLEMENTARY MATERIAL

**Supplement 1.** Primers used for the *ACSS2*.

**Supplement 2.** Sequence information of *ACSS2* gene from the NCBI database.

**Supplement 3.** PCR amplicon of buffalo *ACSS2*.

**Supplement 4.** The CDS of buffalo *ACSS2* and its encoded amino acids.

**Supplement 5.** Basic Physicochemical Characteristics of ACSS2 from buffalo and other mammalian species.

**Supplement 6.** Putative functional modification sites of buffalo ACSS2.

**Supplement 7.** Structural information of the *ACSS2* transcriptional region.

**Supplement 8.** Amino acid sequence identity of ACSS2 in buffalo and other mammals.

**Supplement 9.** Phylogenetic relationships, motifs, and conserved domains of ACSS2 in buffalo and other mammals.

**Supplement 10.** Secondary structure of ACSS2 in buffalo and other Bovidae species.

**Supplement 11.** Secondary structure composition of buffalo and other Bovidae species ACSS2.

**Supplement 12.** The three-dimensional structure of buffalo ACSS2 and its homologues in other mammals.

**Supplement 13.** Evaluation of two pairs of siRNAs for *ACSS2* interference efficiency in BuMECs.
