# Supplementary material for: ACSS2 governs milk fat synthesis in buffalo via a reciprocal positive feedback loop with SREBP1 and PPARG
Source: Anim Biosci. 2026 Mar 11;39(6):250642. doi: 10.5713/ab.250642 (PMC13243924; doi:10.5713/ab.250642)
Supplement: Supplementary file 1 [file ab-250642-Supplementary-1.pdf]

Supplement 1. Primers used for the *ACSS2*

**Gene**	**Serial number of template**	**Prime sequences（5'→3'）**	**Product length/bp**	**Annealing temperature/℃**	**Purpose**
ACSS2	XM_044927710	F: GCGGCTACTGTGTTCCGAACT<br>R: GCCAAGCAGGGGGTAATC	2214	59.8	gene isolation
ACSS2	KJ472108	F: CGAGAGAGTTTTGGGGAG<br>R: ATGTGATCTGGGTGGTTT	240	57	mRNA expression detection
ACTB	NM_001290932	F: TCTTGGTCACCTCGTCGTC<br>R: GGCGCGATGATCTTGAT	145	59.5	internal reference
GAPDH	XM_006065800	F: ATGGAGAAGGCTGGGGCTCA<br>R: GCAGGAGGCATTGCTGACAA	144	60	internal reference
RPS23	XM_006059350	F: ACCGACGAGACCAGAAGT<br>R: CTCCAGGAATGTCACCAA	306	60	internal reference
siRNA1-ACSS2	KJ472108	F: UUCUCCGAACGUGUCACGUTT<br>R: ACGUGACACGUUCGGAGAATT	/	/	gene interference
siRNA2-ACSS2	KJ472108	F: CCAUUCCUAUGUCACCUAUTT<br>R: AUAGGUGACAUAGGAAUGGTT	/	/	gene interference
siRNA-NC	/	F: UUCUCCGAACGUGUCACGUTT<br>R: ACGUGACACGUUCGGAGAATT	/	/	negative control
CDK2	XM_006075063	F: TCATCGAGTCCTGCACCGAGA<br>R: AACCAAGGCTCCATATGTCC	212	57	mRNA expression detection
CDK4	XM_006077918	F: TGCCAACTGCATCGTTCACC<br>R: GATACAGCCAACGCTCCAC	214	57	mRNA expression detection
CCNE1	XM_025268989	F: AGCCTAAAATGCGAGCAA<br>R: CATAAGCAAACTGGTGCAAC	225	53.5	mRNA expression detection
CCND1	XM_006072357	F: CCAACCTCCTCAACGACCGAGT<br>R: TTTTCACGGGCTCCAGCGACA	228	61	mRNA expression detection
SREBF1	XM_025280442	F: GCACCGAGGCCAAGTTGAATAA<br>R: CAGGTCCTTCAGCGATTTGCTT	146	57	mRNA expression detection
PPARG	NM_001290893	F: GCTCCAAGAGTACCAAAGTG<br>R: GTCCTCCTGAAGAAACCCTT	204	53.7	mRNA expression detection
FASN	XM_006061793	F: AGGCCAGCTCCGAAGGCAACA<br>R: TACCACGTCGGCCACTTGTGTC	209	64.3	mRNA expression detection
ACACA	XM_025281124	F: CCTCTTCAGACAGGTTCAAGC<br>R: TTCACCGCACACTGTTCCA	234	55	mRNA expression detection
SCD	NM_001290915	F: CGTGCCGTGGTATCTGTGG<br>R: AAAGGTGTGGTGGTAGTTGTGG	217	56.2	mRNA expression detection
DGAT1	NM_001290902	F: ACAGACAAGGACGGAGACG<br>R: CCACAATGACCAGGCACA	268	55	mRNA expression detection
INSIG1	NM_001290924	F: ACGTTCAGCTCTCCTTGACATT<br>R: CTGTCGTCCTATGTTTCCCAC	239	55	mRNA expression detection
CD36	NM_001290838	F: CTTACAATAATACTGCAGATG<br>R: AAGGTGGAAATGAGGCTG	162	55	mRNA expression detection
