# Supplementary material for: ACSS2 governs milk fat synthesis in buffalo via a reciprocal positive feedback loop with SREBP1 and PPARG
Source: Anim Biosci. 2026 Mar 11;39(6):250642. doi: 10.5713/ab.250642 (PMC13243924; doi:10.5713/ab.250642)
Supplement: Supplementary file 2 [file ab-250642-Supplementary-2.pdf]

Supplement 2. Sequence information of *ACSS2* gene from the NCBI database

Species	Accession number of nucleotide sequences	Accession number of protein sequences	CDS length (bp)	CDS status
Buffalo_X1	XM_006048084.4	XP_006048146.3	2142	Complete
Buffalo_X2	XM_006048085.4	XP_006048147.3	2103	Complete
Buffalo_X3	XM_044927710.2	XP_044783645.2	2052	Complete
Cattle	NM_001105339.1	NP_001098809.1	2103	Complete
Cattle_X1	XM_005214586.3	XP_005214643.1	2142	Complete
Cattle_X2	XM_010811409.3	XP_010809711.1	2052	Complete
Cattle_X3	XM_025000407.1	XP_024856175.1	2013	Complete
Bison	XM_010854888.1	XP_010853190.1	2142	Complete
Yak_X1	XM_005900428.2	XP_005900490.1	2142	Complete
Yak_X2	XM_005900429.2	XP_005900491.1	2103	Complete
Zebu_X1	XM_019972303.1	XP_019827862.1	2103	Complete
Zebu_X2	XM_019972304.1	XP_019827863.1	2142	Complete
Goat_X1	XM_005688483.3	XP_005688540.2	2142	Complete
Goat_X2	XM_018057750.1	XP_017913239.1	2103	Complete
Goat_X3	XM_018057751.1	XP_017913240.1	2052	Complete
Sheep_X1	XM_004014514.5	XP_004014563.2	2142	Complete
Sheep_X2	XM_004014513.5	XP_004014562.2	2103	Complete
Horse_X1	XM_003363913.4	XP_003363961.1	2157	Complete
Horse_X2	XM_001501341.4	XP_001501391.1	2118	Complete
Deer_X1	XM_043883293.1	XP_043739228.1	2142	Complete
Deer_X2	XM_043883294.1	XP_043739229.1	2103	Complete
Camel_X1	XM_010975157.2	XP_010973459.1	2148	Complete
Camel_X2	XM_010975158.2	XP_010973460.1	2109	Complete
Human	NM_018677.4	NP_061147.1	2103	Complete
Rat	NM_001107793.1	NP_001101263.1	2103	Complete
