# Supplementary figures and images for: ACSS2 governs milk fat synthesis in buffalo via a reciprocal positive feedback loop with SREBP1 and PPARG
Source: Anim Biosci. 2026 Mar 11;39(6):250642. doi: 10.5713/ab.250642 (PMC13243924; doi:10.5713/ab.250642)

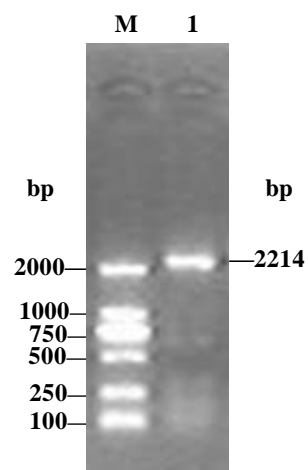

**Supplement 3.** PCR amplicon of buffalo *ACSS2*. M, DL2000 DNA Marker; 1, PCR product.

Supplement: Supplementary file 3 [file ab-250642-Supplementary-3.pdf]
