# Supplementary material for: ACSS2 governs milk fat synthesis in buffalo via a reciprocal positive feedback loop with SREBP1 and PPARG
Source: Anim Biosci. 2026 Mar 11;39(6):250642. doi: 10.5713/ab.250642 (PMC13243924; doi:10.5713/ab.250642)
Supplement: Supplementary file 4 [file ab-250642-Supplementary-4.pdf]

```
1    ATGGGACTTCCCGAAGAGCGGAGCCGGAGCGGCAGCGGGAGCGGGGCCCGGGAGGAGGTTGGAGCCCGGAGCCCGACACGGAGCTGGTCTCCGCCGCCCGAGGTCAGCCGTTCGGCGCAT
1     M  G  L  P  E  E  R  S  R  S  G  S  G  S  G  A  R  E  E  V  G  A  R  S  P  T  R  S  W  S  P  P  P  E  V  S  R  S  A  H
121  GTCCCCTCGCTGCAGCGCTACCGCGAGCTGCACCGGCGCTCCTTGGAGGAGCCGAGAGAGTTTTGGGGAGACATTGCCAAGGAATTTTACTGGAAGACCCCATGTCCTGGTCCATTCCTT
41    V  P  S  L  Q  R  Y  R  E  L  H  R  R  S  L  E  E  P  R  E  F  W  G  D  I  A  K  E  F  Y  W  K  T  P  C  P  G  P  F  L
241  CAGTACAACTTTGATGTGACTAAAGGGAAAATCTTCATTGAATGGATGAAAGGAGCAACTACCAACATCTGCTACAACGTACTGGATCGAATTGTCCATGAGAAAAAACTTGGTGATAAA
81    Q  Y  N  F  D  V  T  K  G  K  I  F  I  E  W  M  K  G  A  T  T  N  I  C  Y  N  V  L  D  R  I  V  H  E  K  K  L  G  D  K
361  GTTGCTTTTTACTGGGAGGGCAATGAGCCAGAGGAAACCACCCAGATCACATACCGAGAGCTTCTGGTCCAAGTGTGTCGGTTCAGCAATGTTCTCCGAAAACAGGGCATTTGCAAGGGT
121   V  A  F  Y  W  E  G  N  E  P  E  E  T  T  Q  I  T  Y  R  E  L  L  V  Q  V  C  R  F  S  N  V  L  R  K  Q  G  I  C  K  G
481  GACCGAGTGGCCATCTACATGCCCATGATCCCGGAGCTTGTGGTGGCTATGCTGGCATGTGCCCGCCTTGGGGCTCTGCACTCCATTGTGTTTGCGGGATTCTCTTCAGAGTCTCTTTGT
161   D  R  V  A  I  Y  M  P  M  I  P  E  L  V  V  A  M  L  A  C  A  R  L  G  A  L  H  S  I  V  F  A  G  F  S  S  E  S  L  C
601  GAACGGATCCTGGATTCCAATTGTAGCCTTCTCATCACTACAGATGCCTTCTACAGGGGGGAAAAGCTTGTCAACCTGAAGGAGCTGGCTGACGAGGCCCTGGAGAAGTGTCAGGAGAAG
201   E  R  I  L  D  S  N  C  S  L  L  I  T  T  D  A  F  Y  R  G  E  K  L  V  N  L  K  E  L  A  D  E  A  L  E  K  C  Q  E  K
721  GGTTCCCCAGTGAAATGCTGCATTGTGGTCAAGCACCTGGGGCGGGCAGAGCTGGGCACAGGTGACTCTCCCAGCCAGTCTCCTCCGATTAAGAGGCCATGCCCGGATGTGCAGATCTCC
241   G  S  P  V  K  C  C  I  V  V  K  H  L  G  R  A  E  L  G  T  G  D  S  P  S  Q  S  P  P  I  K  R  P  C  P  D  V  Q  I  S
841  TGGAATGAGGGGGTTGACTTGTGGTGGCATGAACTCATGCAGAAGGCAGGGGATGAGTGTGAGCCTGAATGGTGTGATGCTGAAGACCCACTCTTCATCCTGTATACCAGTGGCTCCACA
281   W  N  E  G  V  D  L  W  W  H  E  L  M  Q  K  A  G  D  E  C  E  P  E  W  C  D  A  E  D  P  L  F  I  L  Y  T  S  G  S  T
961  GGCAAACCCAAGGGCGTGGTACACACGGTTGGGGGCTACATGCTCTATGTGGCCACAACCTTCAAGTATGTGTTTGACTTTCATGCGGAGGATGTGTTCTGGTGCACCGCAGACATTGGC
321   G  K  P  K  G  V  V  H  T  V  G  G  Y  M  L  Y  V  A  T  T  F  K  Y  V  F  D  F  H  A  E  D  V  F  W  C  T  A  D  I  G
1081 TGGATCACTGGCCATTCCTATGTCACCTATGGGCCACTGGCCAATGGTGCCACCAGTGTTTTGTTTGAGGGGATTCCCACATACCCGGATGTGAGCCGCCTATGGAATATCGTGGAGAAG
361   W  I  T  G  H  S  Y  V  T  Y  G  P  L  A  N  G  A  T  S  V  L  F  E  G  I  P  T  Y  P  D  V  S  R  L  W  N  I  V  E  K
1201 TACAAGGTGACCAAGTTCTACACAGCACCTACAGCCATCCGTCTGCTCATGAAGTTTGGAGATGAGCCTGTTACCAAGCACAGCCGGGCGTCCTTACAGGTGCTAGGAACTGTGGGCGAA
401   Y  K  V  T  K  F  Y  T  A  P  T  A  I  R  L  L  M  K  F  G  D  E  P  V  T  K  H  S  R  A  S  L  Q  V  L  G  T  V  G  E
1321 CCTATCAACCCTGAGGCCTGGCTCTGGTACCACCAGGTGGTAGGTGCGCAGCGCTGCCCCATTGTGGACACCTTCTGGCAGACAGAGACAGGTGGCCATATGCTGACCCCACTCCCTGGG
441   P  I  N  P  E  A  W  L  W  Y  H  Q  V  V  G  A  Q  R  C  P  I  V  D  T  F  W  Q  T  E  T  G  G  H  M  L  T  P  L  P  G
1441 GCCATACCCATGAAGCCTGGTTCTGCTACCTTCCCATTCTTCGGGGTAGCTCCTGCGATCCTGAATGAGTCTGGGGAAGAGTTGGAAGGTGAAGCTGAAGGCTATCTGGTGTTCAAGCAG
481   A  I  P  M  K  P  G  S  A  T  F  P  F  F  G  V  A  P  A  I  L  N  E  S  G  E  E  L  E  G  E  A  E  G  Y  L  V  F  K  Q
1561 CCCTGGCCAGGGATCATGCGCACGGTCTATGGGAACCACGAACGCTTTGAGACCACCTACTTTAAGAAGTTCCCCGGGTACTACGTGACAGGAGATGGCTGCCGGCGGGACAAGGATGGC
521   P  W  P  G  I  M  R  T  V  Y  G  N  H  E  R  F  E  T  T  Y  F  K  K  F  P  G  Y  Y  V  T  G  D  G  C  R  R  D  K  D  G
1681 TATTACTGGATCACCGGCAGGATCGATGACATGCTGAACGTATCTGGACACCTGCTGAGCACGGCAGAGGTGGAGTCAGCACTCGTGGAACACAAGGCTGTTGCAGAGGCAGCCGTGGTT
561   Y  Y  W  I  T  G  R  I  D  D  M  L  N  V  S  G  H  L  L  S  T  A  E  V  E  S  A  L  V  E  H  K  A  V  A  E  A  A  V  V
1801 GGCCACCCTCATCCTGTGAAGGGCGAATGCCTCTACTGTTTTGTCACCTTGTGCGATGGCCACATCTTCAGCCCAGCTCTTACTGAGGAGCTCAAGAAGCAGATTAGAGAAAAGATTGGC
601   G  H  P  H  P  V  K  G  E  C  L  Y  C  F  V  T  L  C  D  G  H  I  F  S  P  A  L  T  E  E  L  K  K  Q  I  R  E  K  I  G
1921 CCCATTGCCACACCGGATTACATCCAGAATGCACCTGGATTGCCTAAAACTCGCTCAGGGAAAATCATGAGGCGGGTGCTTCGGAAGATCGCTCAGAATGACCACGACCTGGGGGACACG
641   P  I  A  T  P  D  Y  I  Q  N  A  P  G  L  P  K  T  R  S  G  K  I  M  R  R  V  L  R  K  I  A  Q  N  D  H  D  L  G  D  T
2041 TCCACTGTAGTCGACCCATCCATCATCACCCAGCTCTTCAGCCACCGCTGCTTGACCATCCAATGA
681   S  T  V  V  D  P  S  I  I  T  Q  L  F  S  H  R  C  L  T  I  Q  *
```

Supplement 4. The CDS of buffalo *ACSS2* and its encoded amino acids. *, stop codon. Shaded area, ACS conserved domain.
