# Supplementary material for: ACSS2 governs milk fat synthesis in buffalo via a reciprocal positive feedback loop with SREBP1 and PPARG
Source: Anim Biosci. 2026 Mar 11;39(6):250642. doi: 10.5713/ab.250642 (PMC13243924; doi:10.5713/ab.250642)
Supplement: Supplementary file 5 [file ab-250642-Supplementary-5.pdf]

Supplement 5. Basic Physicochemical Characteristics of ACSS2 from buffalo and other mammalian species

**Basic physicochemical properties**	**Buffalo (this study)**	**Cattle _X1**	**Yak _X2**	**Zebu _X1**	**Goat _X2**	**Sheep _X2**
Number of amino acids	701	701	701	701	701	701
molecular weight(kD)	78.62	78.73	78.73	78.69	78.72	78.79
Isoelectric point	6.03	6.10	6.10	6.03	6.03	6.03
Negatively charged residues (Asp+Glu)	86	86	86	86	86	86
Positively charged residues (Arg+Lys)	75	76	76	75	75	75
Hydrophobic amino acids (A, I, L, F, W, V)	234	236	236	236	236	235
Polar amino acids (N, C, Q, S, T, Y)	167	165	165	165	168	167
Instability index	41.09	40.96	40.96	41.18	41.01	40.53
Grand average of hydropathicity	-0.260	-0.254	-0.254	-0.248	-0.258	-0.263
Aliphatic index	79.39	79.67	79.67	79.67	79.96	79.53
