# Supplementary material for: ACSS2 governs milk fat synthesis in buffalo via a reciprocal positive feedback loop with SREBP1 and PPARG
Source: Anim Biosci. 2026 Mar 11;39(6):250642. doi: 10.5713/ab.250642 (PMC13243924; doi:10.5713/ab.250642)
Supplement: Supplementary file 6 [file ab-250642-Supplementary-6.pdf]

Supplement 6. Putative functional modification sites of buffalo ACSS2

Putative functional sites	Position and amino composition
N-myristoylation sites	11-16:GSgsGA; 98-103:GattNI; 156-161:GickGD; 184-189:GalhSI; 325-330:GVvhTV; 495-500:GvapAI; 524-529:GimrTV; 653-658:GLpkTR
Casein kinase II phosphorylation sites	54-57:SleE; 137-140: TyrE; 198-201: SlcE; 280-283: SwnE; 387-390:TypD; 437-440: TvgE; 504-507: SgeE; 580-583: StaE; 616-619: TlcD; 682-685:TvvD
N-glycosylation sites	207-210:NCSL; 502-505: NESG;573-576: NVSG
Tyrosine kinase phosphorylation site	555-562:RrdkDgy.Y;
Protein kinase C phosphorylation sites	137-139:TyR; 320-322:TgK; 340-342: TfK; 565-567: TgR; 659-661: SgK; 694-696: ShR
