# Supplementary material for: ACSS2 governs milk fat synthesis in buffalo via a reciprocal positive feedback loop with SREBP1 and PPARG
Source: Anim Biosci. 2026 Mar 11;39(6):250642. doi: 10.5713/ab.250642 (PMC13243924; doi:10.5713/ab.250642)
Supplement: Supplementary file 7 [file ab-250642-Supplementary-7.pdf]

Supplement 7. Structural information of the *ACSS2* transcriptional region

Species	Length (bp)																					
	**5' UTR**	**E1**	**E2**	**E3**	**E4**	**E5**	**E6**	**E7**	**E8**	**E9**	**E10**	**E11**	**E12**	**E13**	**E14**	**E15**	**E16**	**E17**	**E18**	**E19**	**E20**	**3' UTR**
Buffalo_X1	78	256	196	92	104	73	76	115	39	138	171	134	133	57	81	109	69	177	75	869		744
Buffalo_X2	76	254	196	92	104	73	76	115		138	171	134	133	57	81	109	69	177	75	869		744
Buffalo_X3	80	258	196	92	104	73	76	115	39	138	171	134	133	57	81	109	69	177	75	895		860
Cattle	49	227	196	92	104	73	76	115		138	171	134	133	57	81	109	69	177	75	873		748
Cattle_X1	77	255	196	92	104	73	76	115	39	138	171	134	133	57	81	109	69	177	75	873		748
Cattle_X2	77	255	196	92	104	73	76	115	39	138	171	134	133	57	81	109	69	177	75	899		864
Cattle_X3	77	255	196	92	104	73	76	115		138	171	134	133	57	81	109	69	177	75	634		599
Bison		178	196	92	104	73	76	115	39	138	171	134	133	57	81	109	69	177	75	873		748
Yak_X1	41	219	196	66	26	104	73	76	115	39	138	171	134	133	57	81	109	69	177	75	873	748
Yak_X2		178	196	66	26	104	73	76	115	138	171	134	133	57	81	109	69	177	75	864		739
Zebu_X1	119	297	196	92	104	73	76	115		138	171	134	133	57	81	109	69	177	75	873		748
Zebu_X2	76	254	196	92	104	73	76	115	39	138	171	134	133	57	81	109	69	177	75	873		748
Goat_X1	112	290	196	92	104	73	76	115	39	138	171	134	133	57	81	109	69	177	75	879		754
Goat_X2	112	290	196	92	104	73	76	115		138	171	134	133	57	81	109	69	177	75	879		754
Goat_X3	88	266	196	92	104	73	76	115	39	138	171	134	133	57	81	109	69	177	75	905		870
Sheep_X1	66	244	196	92	104	73	76	115	39	138	171	134	133	57	81	109	69	177	75	879		754
Sheep_X2	66	244	196	92	104	73	76	115		138	171	134	133	57	81	109	69	177	75	879		754
Horse_X1	218	411	196	92	104	73	76	115	39	138	171	134	133	57	81	109	69	177	75	880		755
Horse_X2		193	196	92	104	73	76	115		138	171	134	133	57	81	109	69	177	75	880		755
Deer_X1	79	257	196	92	104	73	76	115	39	138	171	134	133	57	81	109	69	177	75	873		748
Deer_X2	79	257	196	92	104	73	76	115		138	171	134	133	57	81	109	69	177	75	873		748
Camel_X1	75	259	196	92	104	73	76	115	39	138	171	134	133	57	81	109	69	177	75	872		747
Camel_X2	73	257	196	92	104	73	76	115		138	171	134	133	57	81	109	69	177	75	869		744
Human	34	212	196	92	104	73	76	115		138	171	134	133	57	81	109	69	177	75	873		748
Rat	64	242	196	92	104	73	76	115		138	171	134	133	57	81	109	69	177	75	825		700

“E” stands for “Exon”.
