# Supplementary material for: ACSS2 governs milk fat synthesis in buffalo via a reciprocal positive feedback loop with SREBP1 and PPARG
Source: Anim Biosci. 2026 Mar 11;39(6):250642. doi: 10.5713/ab.250642 (PMC13243924; doi:10.5713/ab.250642)
Supplement: Supplementary file 8 [file ab-250642-Supplementary-8.pdf]

Percent Similarity

Percent Divergence

	1	2	3	4	5	6	7	8	9	10	11	12	13	14	15	16	17	18	19	20	21	22	23		
**1**		99.3	99.3	98.1	98.1	99.1	99.3	99.3	99.1	99.1	98.6	98.6	97.5	98.9	98.9	94.6	94.6	98.6	98.6	95.4	95.4	93.9	92.4	**1**	Buffalo_AIM41258.1
**2**	0.7		100.0	98.8	98.8	99.9	100.0	100.0	99.9	99.9	98.4	98.4	97.3	98.7	98.7	94.4	94.4	98.4	98.4	95.1	95.1	94.0	92.4	**2**	Cattle_NM_001105339.1_X1
**3**	0.7	0.0		98.8	98.8	99.9	100.0	100.0	99.9	99.7	98.5	98.4	97.4	98.7	98.7	94.1	94.3	98.3	98.4	94.8	95.1	94.0	92.4	**3**	Cattle_XM_005214586.3_X2
**4**	2.0	1.2	1.2		100.0	98.7	98.8	98.8	98.7	98.5	97.4	97.3	98.5	97.7	97.6	93.3	93.4	97.2	97.3	93.9	94.2	93.3	91.5	**4**	Cattle_XM_010811409.3_X3
**5**	2.0	1.2	1.2	0.0		98.7	98.8	98.8	98.7	98.7	97.3	97.3	98.5	97.6	97.6	93.6	93.6	97.3	97.3	94.2	94.2	93.3	91.5	**5**	Cattle_XM_025000407.1_X4
**6**	0.9	0.1	0.1	1.3	1.4		99.9	99.9	99.7	99.6	98.3	98.3	97.2	98.6	98.6	94.0	94.2	98.2	98.3	94.7	95.0	93.9	92.3	**6**	Bison_XM_010854888.1
**7**	0.7	0.0	0.0	1.2	1.2	0.1		100.0	99.9	99.7	98.5	98.4	97.4	98.7	98.7	94.1	94.3	98.3	98.4	94.8	95.1	94.0	92.4	**7**	Yak_XM_005900428.2_X1
**8**	0.7	0.0	0.0	1.2	1.2	0.1	0.0		99.9	99.9	98.4	98.4	97.3	98.7	98.7	94.4	94.4	98.4	98.4	95.1	95.1	94.0	92.4	**8**	Yak_XM_005900429.2_X2
**9**	0.9	0.1	0.1	1.4	1.4	0.3	0.1	0.1		100.0	98.4	98.4	97.3	98.7	98.7	94.3	94.3	98.3	98.3	95.0	95.0	94.0	92.3	**9**	Zebu_XM_019972303.1_X1
**10**	0.9	0.1	0.3	1.5	1.4	0.4	0.3	0.1	0.0		98.3	98.4	97.2	98.6	98.7	93.8	94.2	98.0	98.3	94.7	95.0	94.0	92.3	**10**	Zebu_XM_019972304.1_X2
**11**	1.4	1.6	1.6	2.7	2.7	1.7	1.6	1.6	1.6	1.7		100.0	98.8	99.3	99.3	93.7	93.9	97.9	98.0	94.5	94.9	93.6	91.9	**11**	Goat_XM_005688483.3_X1
**12**	1.4	1.6	1.6	2.7	2.7	1.7	1.6	1.6	1.6	1.6	0.0		98.8	99.3	99.3	94.0	94.0	98.0	98.0	94.9	94.9	93.6	91.9	**12**	Goat_XM_018057750.1_X2
**13**	2.6	2.7	2.7	1.5	1.5	2.8	2.7	2.7	2.7	2.8	1.2	1.2		98.1	98.1	93.0	93.1	96.9	97.0	93.7	94.0	93.0	91.1	**13**	Goat_XM_018057751.1_X3
**14**	1.2	1.3	1.3	2.4	2.4	1.4	1.3	1.3	1.3	1.4	0.7	0.7	1.9		100.0	93.8	94.0	98.2	98.3	94.5	94.9	93.7	92.3	**14**	Sheep_XM_004014514.5_X1
**15**	1.2	1.3	1.3	2.4	2.4	1.4	1.3	1.3	1.3	1.3	0.7	0.7	2.0	0.0		94.2	94.1	98.3	98.3	94.9	94.9	93.7	92.3	**15**	Sheep_XM_004014513.5_X2
**16**	5.6	5.8	6.1	7.1	6.7	6.3	6.1	5.8	5.9	6.4	6.6	6.3	7.4	6.4	6.1		99.9	93.8	94.3	95.1	95.3	94.2	93.0	**16**	Horse_XM_003363913.4_X1
**17**	5.7	5.8	5.9	6.9	6.7	6.1	5.9	5.8	6.0	6.1	6.4	6.3	7.2	6.3	6.1	0.1		94.2	94.3	95.2	95.3	94.1	93.0	**17**	Horse_XM_001501341.4_X2
**18**	1.4	1.6	1.7	2.8	2.7	1.8	1.7	1.6	1.7	2.0	2.1	2.0	3.1	1.8	1.7	6.4	6.1		100.0	94.4	94.9	93.9	92.6	**18**	Camel_XM_010975157.2_X1
**19**	1.4	1.6	1.6	2.7	2.7	1.7	1.6	1.6	1.7	1.7	2.0	2.0	3.0	1.7	1.7	5.9	6.0	0.0		94.9	94.9	93.9	92.6	**19**	Camel_XM_010975158.2_X2
**20**	4.7	5.0	5.4	6.4	6.1	5.5	5.4	5.0	5.2	5.5	5.7	5.3	6.6	5.7	5.3	5.1	5.0	5.8	5.3		100.0	94.2	93.4	**20**	Deer_XM_043883293.1_X1
**21**	4.7	5.0	5.0	6.1	6.1	5.2	5.0	5.0	5.2	5.2	5.3	5.3	6.2	5.3	5.3	4.9	4.9	5.3	5.3	0.0		94.2	93.4	**21**	Deer_XM_043883294.1_X2
**22**	6.4	6.3	6.3	7.0	7.0	6.4	6.3	6.3	6.3	6.3	6.7	6.7	7.4	6.6	6.6	6.1	6.1	6.4	6.4	6.1	6.1		93.7	**22**	Human_NM_018677.4
**23**	8.0	8.0	8.0	9.0	9.0	8.1	8.0	8.0	8.1	8.1	8.6	8.6	9.5	8.1	8.1	7.4	7.4	7.8	7.8	6.9	6.9	6.6		**23**	Rat_NM_001107793.1
	**1**	**2**	**3**	**4**	**5**	**6**	**7**	**8**	**9**	**10**	**11**	**12**	**13**	**14**	**15**	**16**	**17**	**18**	**19**	**20**	**21**	**22**	**23**		

Supplement 8. Amino acid sequence identity of ACSS2 in buffalo and other mammals.
