# Supplementary material for: ACSS2 governs milk fat synthesis in buffalo via a reciprocal positive feedback loop with SREBP1 and PPARG
Source: Anim Biosci. 2026 Mar 11;39(6):250642. doi: 10.5713/ab.250642 (PMC13243924; doi:10.5713/ab.250642)
Supplement: Supplementary file 9 [file ab-250642-Supplementary-9.pdf]

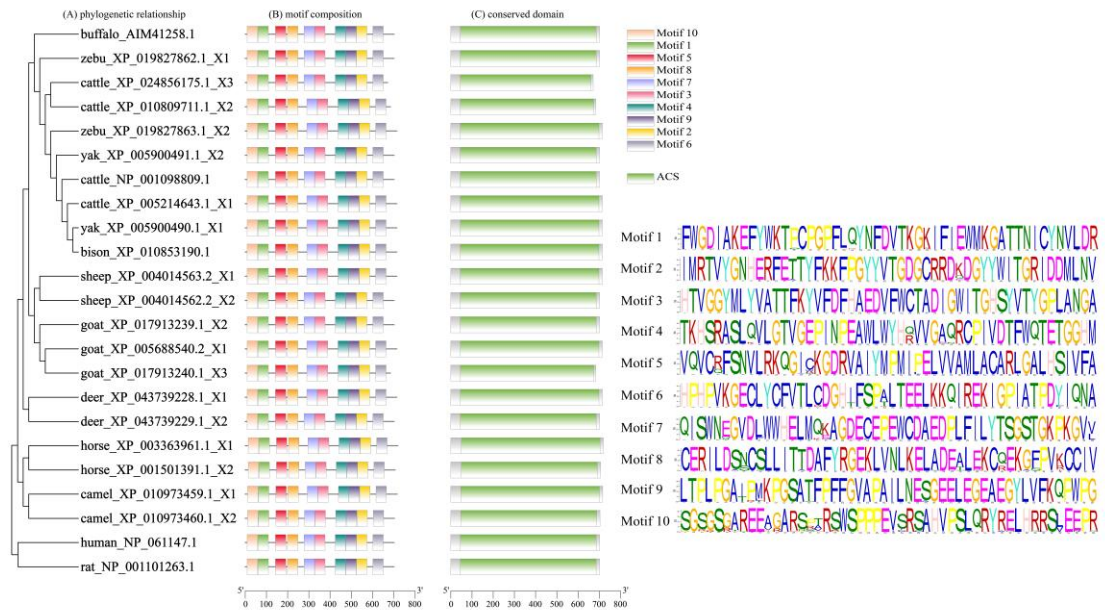

Supplement 9. Phylogenetic relationships, motifs, and conserved domains of ACSS2 in buffalo and other mammals.
