# Supplementary material for: ACSS2 governs milk fat synthesis in buffalo via a reciprocal positive feedback loop with SREBP1 and PPARG
Source: Anim Biosci. 2026 Mar 11;39(6):250642. doi: 10.5713/ab.250642 (PMC13243924; doi:10.5713/ab.250642)
Supplement: Supplementary file 10 [file ab-250642-Supplementary-10.pdf]

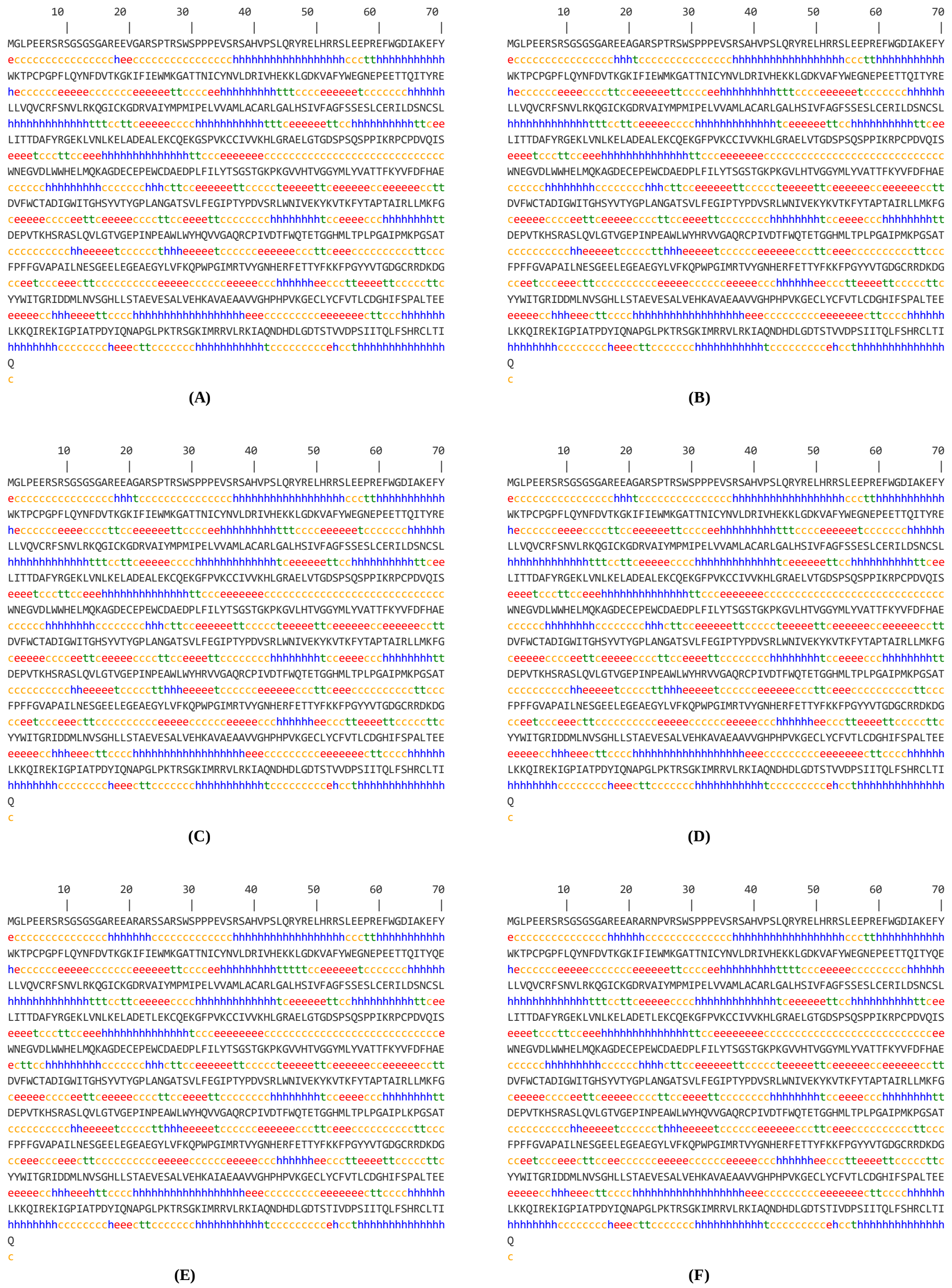

Supplement 10. Secondary structure of ACSS2 in buffalo and other Bovidae species. h, e, t and c represent α-helix, extension chain, β turn and random coil, respectively. (A) buffalo (AIM41258.1); (B) cattle (NP_001098809.1); (C) yak (XP_005900491.1); (D) zebu (XP_019827862.1); (E) goat (XP_017913239.1); (F) sheep (XP_004014562.2).
