# Supplementary material for: ACSS2 governs milk fat synthesis in buffalo via a reciprocal positive feedback loop with SREBP1 and PPARG
Source: Anim Biosci. 2026 Mar 11;39(6):250642. doi: 10.5713/ab.250642 (PMC13243924; doi:10.5713/ab.250642)
Supplement: Supplementary file 11 [file ab-250642-Supplementary-11.pdf]

Supplement 11. Secondary structure composition of buffalo and other Bovidae species ACSS2

**Structer**	**Buffalo (this study)**	**Cattle _X1**	**Yak _X2**	**Zebu _X1**	**Goat _X2**	**Sheep _X2**
Random coil (%)	40.23	40.23	40.23	40.23	38.80	39.37
Alpha helix (%)	27.96	28.25	28.25	28.25	29.10	28.96
Beta turn (%)	9.56	9.84	9.84	9.84	9.70	9.27
Extended strand (%)	22.25	21.68	21.68	21.68	22.40	22.40
