# Supplementary material for: ACSS2 governs milk fat synthesis in buffalo via a reciprocal positive feedback loop with SREBP1 and PPARG
Source: Anim Biosci. 2026 Mar 11;39(6):250642. doi: 10.5713/ab.250642 (PMC13243924; doi:10.5713/ab.250642)
Supplement: Supplementary file 12 [file ab-250642-Supplementary-12.pdf]

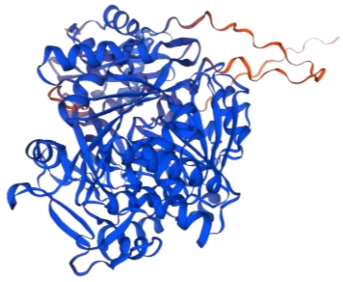

(A)

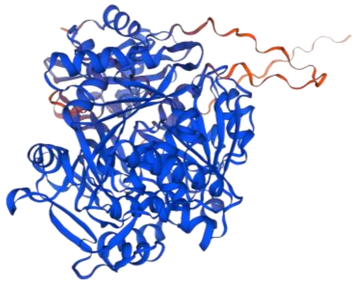

(B)

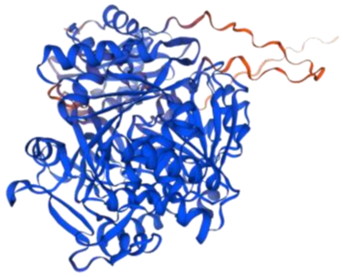

(C)

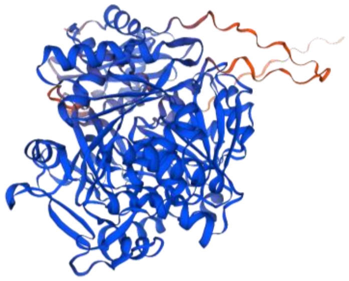

(D)

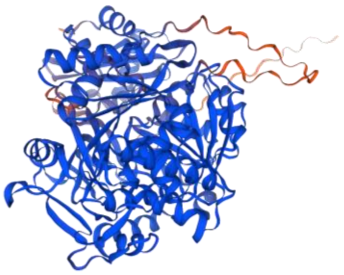

(E)

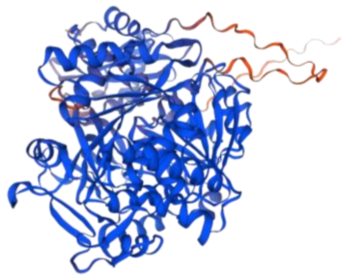

(F)

Supplement 12. The three-dimensional structure of buffalo ACSS2 and its homologues in other mammals. The coverage of buffalo (AIM41258.1), cattle (NP_001098809.1), yak (XP_005900491.1), zebu (XP_019827862.1), goat (XP_017913239.1) and sheep (XP_004014562.2) ACSS2 protein sequences with the template (Q9NR19.1.A) sequence were 100%, and the sequence identity was 93.87% , 94.01%, 94.01%, 94.01%, 93.58% and 93.72%, respectively. (A) buffalo (AIM41258.1); (B) cattle (NP_001098809.1); (C) yak (XP_005900491.1); (D) zebu (XP_019827862.1); (E) goat (XP_017913239.1); (F) sheep (XP_004014562.2).
