# Supplementary material for: ACSS2 governs milk fat synthesis in buffalo via a reciprocal positive feedback loop with SREBP1 and PPARG
Source: Anim Biosci. 2026 Mar 11;39(6):250642. doi: 10.5713/ab.250642 (PMC13243924; doi:10.5713/ab.250642)
Supplement: Supplementary file 13 [file ab-250642-Supplementary-13.pdf]

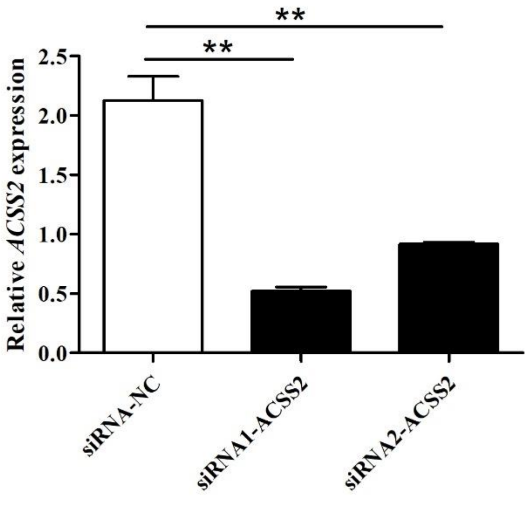

Supplement 13. Evaluation of two pairs of siRNAs for *ACSS2* interference efficiency in BuMECs. The values are presented as means ± SEM; *, $p < 0.05$; **, $p < 0.01$.
